# Design and Characterization of a Peptide Mimotope of the HIV-1 gp120 Bridging Sheet

**DOI:** 10.3390/ijms13055674

**Published:** 2012-05-10

**Authors:** Marco Schiavone, Giuseppe Fiume, Antonella Caivano, Annamaria de Laurentiis, Cristina Falcone, Francesca Fasanella Masci, Enrico Iaccino, Selena Mimmi, Camillo Palmieri, Antonio Pisano, Marilena Pontoriero, Annalisa Rossi, Annarita Scialdone, Eleonora Vecchio, Concetta Andreozzi, Maria Trovato, Jan Rafay, Boris Ferko, David Montefiori, Angela Lombardi, Giulia Morsica, Guido Poli, Ileana Quinto, Vincenzo Pavone, Piergiuseppe de Berardinis, Giuseppe Scala

**Affiliations:** 1 Department of Clinical and Experimental Medicine, University of Catanzaro “Magna Graecia”, Catanzaro 88100, Italy; E-Mails: annadela@unicz.it (A.L.); cfalcone@unicz.it (C.F.); fasanellamasci@unicz.it (F.F.M.); iaccino@unicz.it (E.I.); selena.mimmi@gmail.com (S.M.); cpalmieri@unicz.it (C.P.); pisano@unicz.it (A.P.); pontoriero@unicz.it (M.P.); rossia@unicz.it (A.R.); scialdone@unicz.it (A.S.); eleonoravecchio@unicz.it (E.V.); 2 Department of Biochemistry and Medical Biotechnology, University of Naples “Federico II”, Naples 80131, Italy; 3 IBP-CNR, Naples 80131, Italy; E-Mails: acaivano@ibp.cnr.it (A.C.); mtrovato@ibp.cnr.it (M.T.); deberardinis@ibp.cnr.it (P.B.); 4 Department of Chemistry, University of Naples “Federico II”, Naples 80131, Italy; E-Mails: concetta.andreozzi@unina.it (C.A.); angelina.lombardi@unina.it (A.L.); vincenzo.pavone@unina.it (V.P.); 5 Research Institute of Animal Production, Nitra 94992, Slovakia; E-Mail: rafay@cvzv.sk; 6 Institute of Applied Microbiology, Wien A-1190, Austria; E-Mail: b.ferko@greenhillsbiotech.com; 7 Laboratory for AIDS Vaccine Research & Development, Department of Surgery, Duke University Medical Center, Durham, NC 27710, USA; E-Mail: monte@duke.edu; 8 San Luigi AIDS Centre, San Raffaele Scientific Institute, Milan 20127, Italy; E-Mails: morsica.giulia@hsr.it (G.M.); poli.guido@hsr.it (G.P.); 9 Division of Immunology, Transplantation and Infectious Diseases, Vita-Salute San Raffaele University School of Medicine, Milan 20132, Italy

**Keywords:** HIV-1 vaccine, bridging sheet, mimotope

## Abstract

The Bridging Sheet domain of HIV-1 gp120 is highly conserved among the HIV-1 strains and allows HIV-1 binding to host cells via the HIV-1 coreceptors. Further, the bridging sheet domain is a major target to neutralize HIV-1 infection. We rationally designed four linear peptide epitopes that mimic the three-dimensional structure of bridging sheet by using molecular modeling. Chemically synthesized peptides BS3 and BS4 showed a fair degree of antigenicity when tested in ELISA with IgG purified from HIV^+^ broadly neutralizing sera while the production of synthetic peptides BS1 and BS2 failed due to their high degree of hydrophobicity. To overcome this limitation, we linked all four BS peptides to the COOH-terminus of GST protein to test both their antigenicity and immunogenicity. Only the BS1 peptide showed good antigenicity; however, no envelope specific antibodies were elicited upon mice immunization. Therefore we performed further analyses by linking BS1 peptide to the NH2-terminus of the E2 scaffold from the *Geobacillus Stearothermophylus* PDH complex. The E2-BS1 fusion peptide showed good antigenic results, however only one immunized rabbit elicited good antibody titers towards both the monomeric and oligomeric viral envelope glycoprotein (Env). In addition, moderate neutralizing antibodies response was elicited against two HIV-1 clade B and one clade C primary isolates. These preliminary data validate the peptide mimotope approach as a promising tool to obtain an effective HIV-1 vaccine.

## 1. Introduction

Neutralizing antibodies (Nabs) play a key role in controlling viral infections and contribute to the protective effect of many successful vaccines [1]. In particular, neutralizing antibodies bind viral particles preventing viral infection of host target cells and blocking subsequent replication cycles. Furthermore, neutralizing antibodies could associate to the Fcγ receptor, triggering a cascade of events that has the potential to contain cell-cell HIV-1 spread [2].

The main step toward an effective vaccine against the Human Immunodeficiency Virus-1 (HIV-1) infection relies on the identification of immunogens able to elicit broadly neutralizing responses [3]. Most of type-specific and broadly cross-reactive neutralizing antibodies elicited during HIV-1 natural infection are mainly directed against HIV-1 Env glycoproteins gp120 and gp41, that play a major role for viral attachment and entry into target cells [4]. The initial antibody response to HIV-1 is primarily directed against non-neutralizing epitopes on envelope glycoproteins gp120 and gp41 (Env) [5,6]. These antibodies are unable to control viremia and apparently do not exert any selective immune pressure on the HIV-1 envelope [7]. Months after HIV-1 infection, neutralizing antibodies appear; however they do not show any broadly neutralizing activity against viral isolates (heterologous strain) different from infecting strain (autologous strain) [8–10]. These autologous neutralizing antibodies drive the viral escape occurring through single amino acid substitutions, insertions and deletions, and through an “evolving glycan shield” where shifting glycans prevent access of neutralizing antibodies to their cognate epitopes [1,11]. The neutralizing activity of autologous NAbs is mostly directed toward conformational epitopes such as: the variable regions of gp120, the CD4 binding site and the epitopes located at the *N*- or *C*-terminal residues of gp120 [12]. Most antibodies that target these regions do not neutralize primary isolates effectively, due to the poor accessibility of the structural epitopes in the native oligomeric envelope [13]. The entire native structure of gp120-gp41 complex is unknown; however, extensive information is available on isolated domains of the HIV envelope proteins and their analogues in SIV [13–20]. The X-ray structures of the HIV and SIV gp120 core domain, both in CD4-bound and CD4-unbound state, together with X-ray and NMR studies of gp41 indicate that gp120 and gp41 are non-covalently associated and assembled in trimeric spikes structure [21–23]. The gp120 secondary structure shows a complex refolding: an inner and an outer domain closely connected by the flexible bridging sheet domain (BS), which is a major discontinuous domain on the HIV-1 envelope spike; accordingly, its amino acidic sequence is conserved among different viral clades. The HIV-1 BS domain is composed of four antiparallel β-strands (β2-β3-β20-β21), far from each other in the linear amino acid sequence, and strictly connected in the secondary structure [13]; V1/V2 and V3 loops complete the structure. Accordingly, the BS domain is highly flexible as shown by the many conformational changes observed during the HIV infection [24–26]. In fact, the BS is hidden inside the gp120 core during the initial steps of natural infection, and is briefly exposed only upon the CD4 binding allowing the binding to the CCR5 or CXCR4 co-receptors. As reported, many studies focused on the infection mechanism [13,24–26], have shown that β20-β21 strands undergo a sudden conformational change from β sheet to a partial α-helix structure that binds the CCR5 [12,26–34].

Autologous neutralizing antibodies mainly target the V1/V2 loop of gp120, and to a lesser extent the V4 and V5 loops [35], while it has become clear that anti-V3 antibodies, which are among the first antibodies to be elicited in HIV-1 infection, do not contribute to autologous neutralization [36].

A truly effective vaccine would require broadly neutralizing antibodies (bnAbs) that can protect against a wide array of HIV-1 strains [7].

The first broadly neutralizing human mAb against HIV-1 env, IgGb12, was isolated from a clade B-infected patient in 1992 by phage-displayed techniques. This antibody binds to the CD4-binding site (CD4bs) region of gp120 [37] and can neutralize more than 50% of clade B viral isolates and about 30% of non-clade B viruses [38,39]. Over the years other neutralizing antibodies were identified: one group binds to gp120 (IgG2G12, IgGPG9/PG16, IgGHJ16, IgGVRC01), while another group is specific for gp41 (IgG2F5, IgG4E10 and IgGZ13). Recently, additional broadly neutralizing antibodies have been identified from chronically HIV-1 infected patients. These novel neutralizing antibodies recognize quaternary structures in the context of the native envelope spike and show a high level of somatic mutations, likely owing to an extensive affinity maturation and adaptation, over the course of continuous exposure to an evolving antigen [40–50]. Moreover, broad and potent neutralizing antibodies targeting high-mannose glycan-dependent epitopes of HIV-1 glycoproteins were identified, indicating that glycans are important targets on HIV-1 glycoproteins for broadly neutralizing antibody responses *in vivo*, providing an important lead for future directions in developing NAb-based anti-HIV-1 vaccines [51].

In our work, we used two prokaryotic antigen display systems based on: (a) the GST protein of *Schystosoma japonicum* assessed as good antigen vehicle to the immune system due to the presence of helper peptides endowed into its structure [52]; (b) the acyltransferase component (E2) of the pyruvate dehydrogenase complex from *Geobacillus stearothermophilus.* E2 oligomers form 1.5 MDa 60-mer particles and can display heterologous peptides and proteins [53–56]. E2 60-mer cores can be refolded from denaturing conditions *in vitro* without the help of chaperones [55,56]. Thus, epitopes displayed on the E2 surface elicit both humoral and cellular immune responses [55,56].

We reconstituted a bridging sheet structure by rational design of several peptide mimotope sequences, that were validated using molecular modeling programs matching our peptide mimotopes with the cognate discontinuous epitope on the HIV-1 gp120 envelope glycoprotein in the CD4 binding state [13,16,18,33,57]. All the designed peptide mimotopes were firstly linked to the COOH-terminus of the GST protein. The antigenic and immunogenic properties of GST fusion protein were tested. Due to the ineffective antibody responses in mice, the peptide that better mimicked the bridging sheet was chosen and displayed on the E2 scaffold. We monitored the antigenicity of the bridging sheet mimotope-E2 complex and the ability to elicit in rabbits an antibody response similar to the human immune system. Here, we show that the bridging sheet in a constrained conformation elicits moderate HIV-1 neutralizing antibody titers in one animal.

## 2. Results and Discussion

### 2.1. Rational Design of Bridging Sheet Peptide Mimotopes

**Figure 1:**
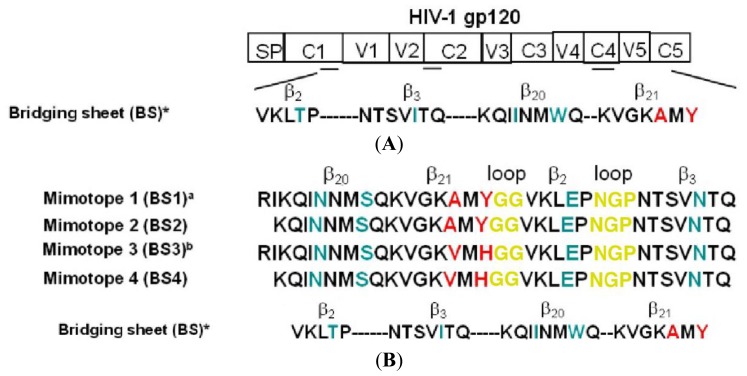

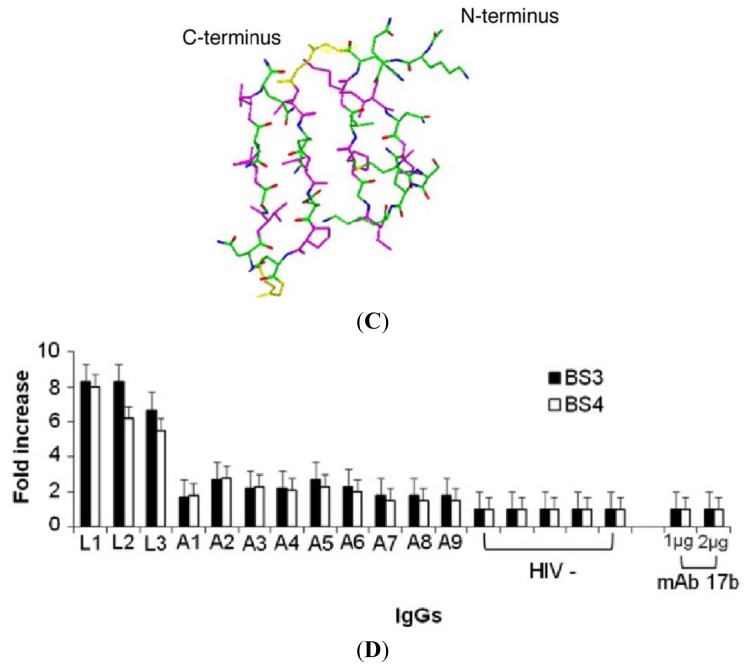
Developing gp120 BS mimotopes and their antigenic characterization. (**A**) Schematic HIV-1 gp120 structure. gp120 is composed of five conserved regions (C1–C5) among different HIV-1 strains and five highly glycosylated hypervariable regions (V1–V5) located in the inner and the outer domain closely connected in 3D structure by a discontinuous bridging sheet structural domain. * The sequence of four Bridging Sheet β strands is reported. (**B**) Mimotope sequences. An extensive bioinformatics analysis was performed to develop Bridging Sheet linear peptide mimotopes. The four β-strands were connected in antiparallel manner beginning from the COOH-terminus β-strands couple (β20 and β21) to the NH-terminus couple (β2 and β3) to resemble the original bridging sheet conformation. Short GG and NGP loops (depicted in yellow) were added for a correct structure refolding. Amino acidic substitutions (depicted in green) were introduced to increase the hydrophilicity and the water solubility of mimotopes. Amino acidic residues essential for CCR5 binding: ^a^ Mimotope 1 and mimotope 2 include the aminoacidic residues essential for CCR5 binding (in red). They were not chemically synthesized but converted in cDNA and directly cloned in prokaryotic expression vectors. ^b^ Mimotope 3 and mimotope 4 were chemically synthesized and the aminoacidic residues essential for CCR5 binding (in red) were substituted to increase the structure solubility. (**C**) Mimotope structure. Molecular modeling programs showed that all bridging sheet mimotopes folded as four antiparallel β-strands structure resembling the original Bridging Sheet conformation. (**D**) Testing reactivity of chemically synthesized peptide mimotopes with a panel of IgGs purified from HIV-1 positive sera. A direct ELISA assay was performed to test the chemically synthesized BS3 (black bars) and BS4 (white bars) reactivity with IgGs purified from LTNPs sera (marked as L1–L3), conclamate AIDS sera (marked as A1–A9) and mAb 17b. HIV-donor sera were used as negative controls. Results are reported as HIV^+^ IgGs/HIV-IgGs ratio (fold increase) and a fold increase ≥3 was a positive result. The graph reports the mean of three independent experiments and the error bars. Student’s *t* test was used to assess the statistical significance of the mimotope reactivity between the LTNP purified IgGs and AIDS purified IgGs (*p* < 0.05 for LTNPs).

We rationally designed peptide mimotopes of the Bridging Sheet three-dimensional structure by using molecular modeling. We took into account some important elements: (1) the bridging sheet is a gp120 discontinuous domain, exposed briefly soon after the CD4 binding, with a complex highly flexible structure [13]. It is composed of four antiparallel β-strands (β2, β3, β20, β21) (Figure 1A) with conserved sequences among different viral strains. These four β-strands are far from each other in the linear amino acidic sequence but strictly connected in 3D Env conformation [13]. (2) The bridging sheet has a double face: a hydrophobic face inside a deep pocket, exposed only after the gp120 binding to the CD4 [13,16,18], making hydrophobic bindings with CCR5 co-receptor only during the early stages of acute infection; an hydrophilic face masked by hyperglycosylated V1/V2 loop and exposed in proximity of CD4 to bind it [13,17,18,24,58]. (3) It is partially recognized by monoclonal, non-neutralizing antibodies directed against CD4i epitopes as mAb 17b [13,16,18] or 4KG5 [18]. After an extensive bioinformatic analysis, we assembled a bridging sheet structure in which four β-strands (β2, β3, β20, β21) sequences were connected in antiparallel manner, beginning from COOH-terminus β-strands couple (β20, β21) to NH-terminus β-strands couple (β2, β3) to mimic the original BS conformation. Correct refolding of the linear mimotopes sequences was obtained by replacing the V1/V2 loop between β2 and β3 with a short NGP loop and adding GG loop between β20 and β21 (Figure 1B). Few amino acid substitutions were introduced into the sequence of chemically synthesized peptide mimotopes BS3 and BS4 to improve their solubility, as A→V and Y→H in β21, I→N and W→S in β20, T→E in β2 and I→N in β3. As A→V and Y→H in β21 are essential for the co-receptor binding, we reproduced two other mimotopes BS1 and BS2 with unmodified β21 strand sequence (Figure 1B). The mimotope BS1 and the mimotope BS3 also included RI amino acidic residues of β19 previously shown to be important for both a correct bridging sheet refolding [24,26] and the improvement of CCR5 binding affinity. Molecular modeling studies with several programs useful to predict the secondary structures of proteins indicated that the 3D structure of peptide mimotopes resembled the original bridging sheet structure during the CD4 binding state (Figure 1C).

### 2.2. Antigenic Properties of Synthetic BS3 and BS4 Peptides

We were unable to obtain chemically synthesized BS1 and BS2 mimoptopes because of their hydrophobicity. Thus, we tested only BS3 and BS4 synthetic peptide mimotopes reactivity with the affinity-purified IgGs from HIV^+^ broadly neutralizing patients’ sera by using a standard ELISA assay. A panel of twelve HIV^+^ sera (3 LTNPs and 9 AIDS) was assessed at fixed 1:1000 dilution. (Figure 1D). All the LTNPs IgGs recognized synthetic peptides; however, a significant difference in reactivity between LTNPs and AIDS IgGs was shown according to standard parametric Student’s *t* test (*p* < 0.05). These data may confirm the inability of the immune system of AIDS patients compared to LTNPs to produce a sustained antibody response against the HIV-1 bridging sheet domain [59,60]. However, the bridging sheet mimotopes were not recognized by the mAb 17b (Figure 1D), a non-neutralizing antibody that recognizes a complex domain containing the bridging sheet. This evidence may be a likely result of the limited physical interaction between the bridging sheet, the mAb 17b and the difficulty to mimic the original conformation of the bridging sheet domain, which also includes few amino acidic residues of V3 loop crown, V3 loop stem and other important residues [13].

### 2.3. Antigenic and Immunogenic Characterization of BS Peptide Mimotopes Linked to GST Protein

**Figure 2:**
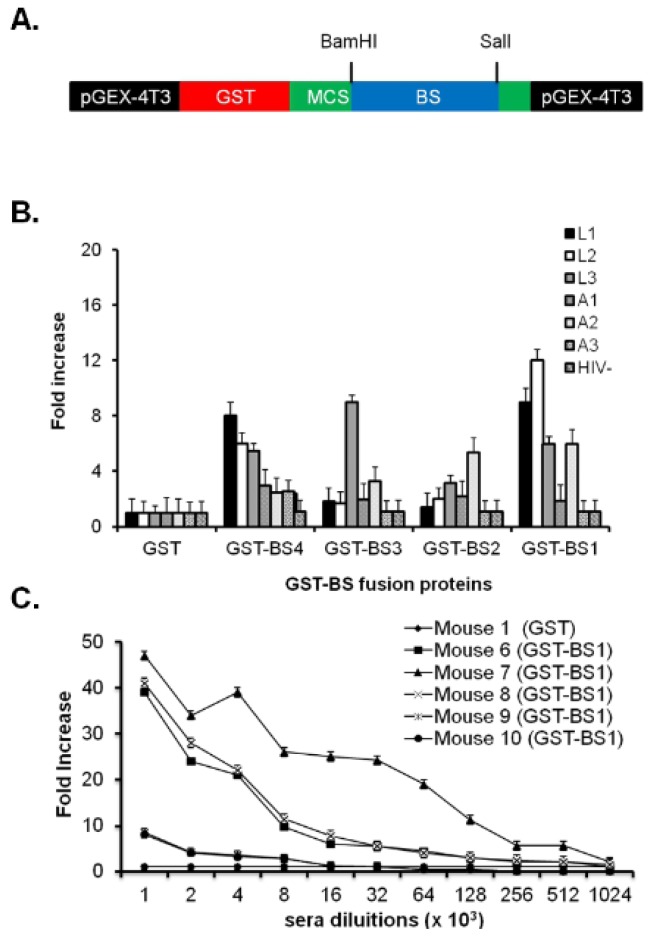
Antigenic and immunogenic characterization of GST-BS1 fusion protein. (**A**) Cloning strategy to link the four Bridging Sheet peptide mimotopes to the COOH-terminus of GST protein. The four Bridging Sheet peptide mimotopes (blue box) were cloned as cDNA into the Multiple Cloning Site (MCS) (green box) of pGEX-4T3 expression plasmid (black boxes) to be linked to the COOH-terminus of GST protein (red box). *Bam*HI and *Sal*I cloning sites are shown. GST-BS fusion proteins were purified as described in [62,63]. (**B**) Antigenicity of four GST-BS fusion proteins. ELISA assays were performed to analyze the reactivity of the four Bridging Sheet peptides as fusion proteins against IgGs purified from LTNP and AIDS patient sera. IgGs purified from a HIV-1 negative donor were used as a control. Results are reported as HIV^+^ IgGs/HIV-IgGs ratio (fold increase) and a fold increase ≥3 was a positive result. The graph reports the mean of five independent experiments and the error bars. According to the Student’s *t* test result obtained by IgGs purified from LTNPs was statistically significant with a *p* < 0.05. (**C**) Mice antibody titers against GST-BS1 fusion protein. Bridging Sheet specific antibody titers were analyzed by performing a direct ELISA assay. The graph shows the results from three independent experiments and the antibody titers obtained immunizing the mouse 7 (black triangle) were statistically significant, by Student’s *t* test, with a *p* < 0.01. The data are reported as GST-BS1 immunized mice/GST immunized mouse (full black rhomb) ratio (fold increase) and sera dilutions with a fold increase ≥3 were positive. Error bars are indicated. The values of sera dilutions on the horizontal axis have to be incremented ×10^3^ as indicated.

Human neutralizing antibodies are able to recognize complex conformational epitopes with a really specific and constrained structure. To constrain the Bridging Sheet mimotopes structure and to increase the low level of solubility of BS1 and BS2, we linked all the four Bridging Sheet peptide mimotopes to the COOH-terminus of GST protein (Figure 2A). GST is known to be a good immunogenic vehicle [52] that contains helper peptides useful to improve immune response stimulating the activity of Antigen Presenting Cells. The reactivity of GST-BS fusion proteins with affinity purified IgGs of LTNP and AIDS patient sera was assessed by an indirect ELISA (Figure 2B). Each GST-BS fusion protein showed a variable degree of reactivity against IgGs but the GST-BS1 reactivity with LTNPs IgGs was higher than the others fusion proteins and statistically significant. Further, the BS1 peptide mimotope contained all the amino acids essential to the CCR5 binding and correct refolding of the Bridging Sheet. For this reason, we used BS1 peptide mimotope for further immunogenic investigations in mice. No reactivity was observed with mAb 17b (data not shown). Two immunization experiments were performed using three groups of five mice: (1) not immunized; (2) immunized with GST vehicle alone; (3) immunized with GST-BS1. The affinity purified IgGs titers were very high both for the GST-BS1 fusion protein as well as for synthesized peptides BS3 and BS4 as shown in Figure 2C. Unfortunately, affinity purified IgGs against GST-BS1 did not crossreact with monomeric or oligomeric HIV-1 gp120, preincubated with monoclonal antibody b12 that mimics CD4 [61].

### 2.4. E2-BS1 Sixty-Mer and Its Antigenic Characterization

**Figure 3:**
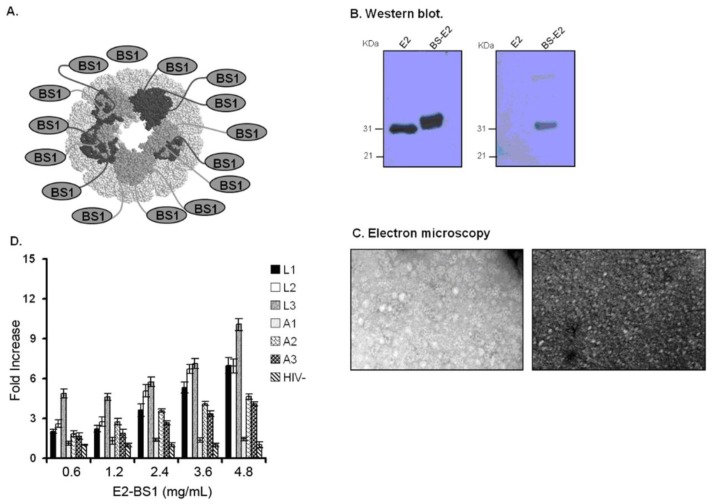
Antigenicity of E2-BS1. (**A**) Schematic representation of *G. stearothermophylus* E2 protein. E2 protein (di-hydro-lypoil-acetyl-transferase) is the central scaffold of the PDH complex. The peptide is directly bound to the core domain of E2 multi-subunit complex without altering the E2 structure. The complex includes about 20 trimeric subunits and each subunit display the BS1 mimotope. (**B**) Western blot analysis to detect E2-BS1 expression. Immunoblot of E2 wt and E2-BS1 recombinant using anti-E2 polyclonal sera (left panel) or anti mouse serum containing antibodies against BS mimotope, obtained from mice immunized by E2-BS1 (right panel). (**C**) Electron microscopy of E2-BS1 complex. Electron micrographs of E2 wild type (left panel) and E2-BS1 recombinant (right panel) stained with uranyl acetate solution. (magnification 160,000×). (**D**) Antigenicity was assessed by performing a direct ELISA assay with increasing concentrations of E2-BS1 protein. IgGs purified from LTNPs sera (marked as L1–L3) and conclamate AIDS sera (marked as A1–A3) were assessed. IgGs purified from a HIV donor serum were used as a control. Results are reported as HIV^+^ sera/HIV serum ratio (fold increase) and a fold increase ≥3 was considered as positive result. The graph reports the mean of three independent experiments and the error bars.

To improve further the antigenicity and the immunogenicity of the BS1 peptide mimotope, which conserved all the residues essential for co-receptor binding in its amino acidic sequence [13,28], we displayed it on the E2 protein scaffold using a prokaryotic expression vector (pETE2DISP) as described in the material and methods section (Figure 3A). E2-BS1 complex was purified from bacterial cells. The NH_2_-terminal sequencing, Western blot analysis using anti E2 and anti BS1 antibodies (Figure 3B) and an Electron Microscopy (Figure 3C) respectively confirmed the correct sequence of the BS1 displayed mimotope, the molecular weight of both the monomeric (about 25 kDa) both the multimeric form (about 120 kDa) and the multimeric structure of E2-BS1 complex. The E2-BS1 complex antigenicity was assessed in ELISA observing the immune reactivity of a complex panel of affinity-purified IgGs from HIV^+^ sera against linear increasing dilutions of E2-BS1 complex. As reported in Figure 3D three representative LTNP IgGs showed better reactivity against the E2-BS1 complex than AIDS IgGs, in a dose dependent manner. The reactivity of E2-BS1 mimotope against the affinity purified IgGs was higher than the reactivity of the synthetic BS3 and BS4 mimotopes demonstrating that a constrained structure can mimic the original antigen more easily.

### 2.5. The Immune Response in Mice and Rabbits

**Figure 4:**
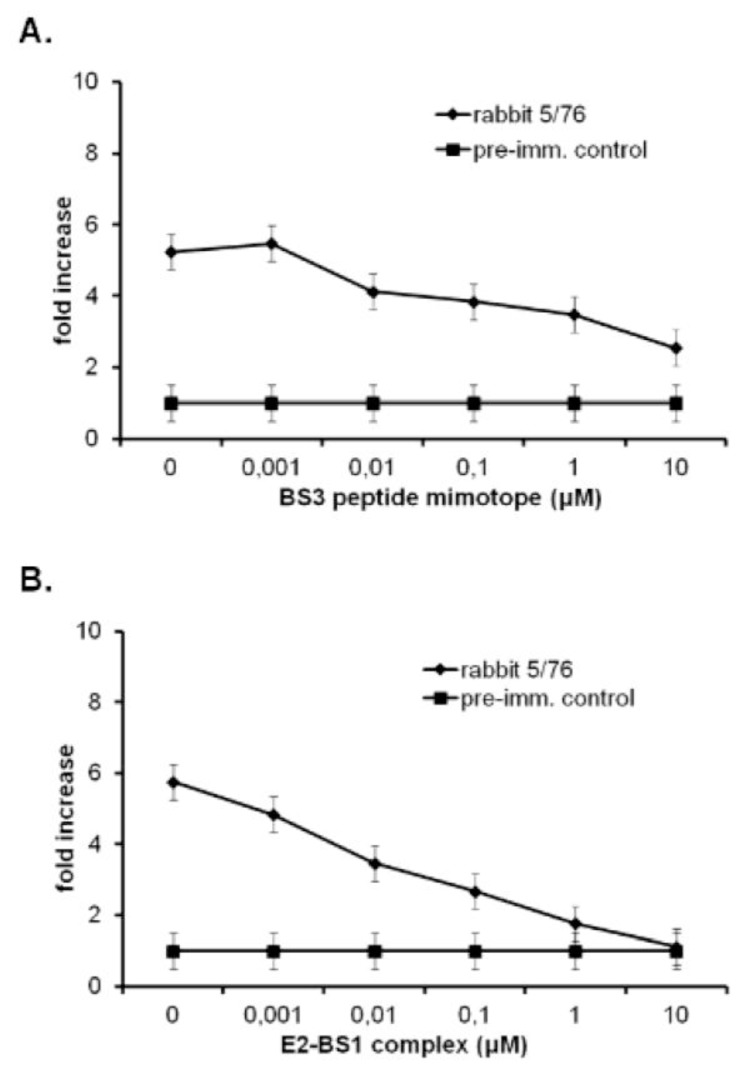
Displacement of m-gp120 SF162 binding. A competitive ELISA assay was performed to analyze the specificity of rabbit immune response against m-gp120 SF162. Rabbit serum 5/76 and its relative pre-immune serum as negative control were incubated with the same increasing molar concentration of BS3 peptide mimotope (**A**) and E2-BS1 complex (**B**). Data were reported as the fold increase of rabbit 5/76/pre-immune serum ratio. The statistical significant was evaluated as the difference in binding displacement between BS3 and E2-BS1, and assessed by performing a standard Student’s *t* test (*p* < 0.05). Error bars were indicated.

To investigate the immunogenic properties of E2-BS1 complex, we immunized i.p. five times at a two week interval two groups of three mice: the first group was immunized with E2-BS1 complex and the second group was immunized with E2 protein alone as a control. The last bleedings showed high IgGs end-point titer (dilution 1:100,000) only against the mimotope (group 1) and the carrier (control group) (Table 1). No response was assessed against either monomeric (m)-gp120 SF162 or oligomeric (o)-gp140ΔV2 SF162 envelope glycoproteins. Moreover, rabbits were immunized in order to generate immunogen specific polyclonal sera endowed with anti-HIV-1 neutralizing activity. We performed two sets of experiments by immunizing two groups of rabbits four times i.m. or s.c. at three weeks intervals; the first group of six rabbits was immunized with E2-BS1 complex, while the control group of four rabbits was immunized with E2 alone. The last bleeding sample was collected two weeks after the fourth immunization to allow the generation of BS1 specific antibodies. As reported in Table 1, only rabbit 5/76 showed a high IgGs end-point titer against the bridging sheet (dilution 1:100,000), together with a detectable cross-reactivity against m-gp120 229 SF162 or gp140ΔV2 SF162 envelope glycoproteins (dilutions 1:5000 and 1:3000 respectively). To confirm the specificity of antibodies directed against envelope glycoproteins, we performed a competitive ELISA by using linear increasing concentrations of the synthetic BS3 mimotope (Figure 4A) and E2-BS1 complex (Figure 4B) as competitors. Both competitors were able to displace the binding between the IgGs from 5/76 rabbit serum and m-gp120 SF162 in a dose dependent manner. Further, the E2-BS1 complex was significantly better than synthetic BS3 peptide as competitor.

### 2.6. Moderate Neutralizing Activity of Antibodies Produced in Rabbits

Once the immunogenic properties of E2-BS1 complex were confirmed, we tested the neutralizing activity of antibodies produced in rabbits. A neutralization assay was performed based on reductions in luciferase (Luc) reporter gene expression after a single round of virus infection with pseudotyped HIV-1 viruses in highly infectious TZM-bl cells [36,64–67], as described in materials and methods section. The neutralizing IgGs activities in rabbit 5/76 (rabbit 5) was assessed against a panel of three highly sensitive heterologous viruses belonging to clades B or C (viruses SF162.LS and NL-ADArs belonging to clade B and one primary clade C HIV-0012466-2.52 isolate). Too mild neutralizing activity against all the three virus strains was seen in two independent neutralization experiments (Table 2A). Just few neutralizing antibodies were detected in rabbit 5 that showed high IgGs titers specific for both the bridging sheet and the viral envelope (Table 1). In particular, the rabbit 5 affinity purified IgGs showed a low neutralization titer against three pseudotyped viruses in the range of 1:30 in the case of the clade C primary isolate and 1:110 to 1:150 in the case of heterologous clade B viruses (Table 2A). No neutralizing antibody titer was assessed in the case of four rabbits immunized with E2-BS1 complex (Table 2B).

## 3. Experimental Section

### 3.1. Ethics Statement

This study was carried out in strict accordance with the recommendations indicated in art.4,5 of D.lgs 116/92 and DD.MM. of 29/09/1995 and 26/04/2000 on animal welfare approved by Italian Ministry of Health and in accordance with the ethical guidelines for animal care of the European Community Council (directive 86/609/ECC). The protocol was approved by the Ethics Committee of University of Catanzaro in accordance with regulation of Italian Ministry of Health, European Community Council (Permit Number: 0008613-P). For the clinical analysis, all of the subjects gave their written informed consent to participate in the study, which was carried out in accordance with the principles expressed in the Declaration of Helsinki. The protocol of the clinical study was approved by the Ethics Committee of San Raffaele Scientific Institute, Milan, Italy in strict accordance with directives C.E. 5/12/2002, C.E. 2/12/04 and C.E. 3/3/2005.

### 3.2. Sera, Env Proteins, Mice, Rabbits

HIV^+^ patient sera (Long Term Non Progressors and full-blown AIDS patients) were obtained from Guido Poli and Giulia Morsica as participants to the Concerted Action “ELVIS” (Evaluation of Long term Non-Progressors Viro-Immunological Italian Studies). Env protein m-gp120 SF162 and o-gp140ΔV2 SF162 were provided by Susan Barnett, Novartis Vaccine and Diagnostics Corporation, Emeryville, CA, USA. Balb/C mice and New Zealand were purchased from Charles River Laboratories International, Wilmington, MA, USA.

### 3.3. Peptide Synthesis

Peptides were chemically synthesized by solid phase methodologies, using Fmoc chemistry. The *N*- and *C*-termini were acetylated and amidated respectively. Peptides were then purified by reverse phase HPLC to >90% homogeneity and confirmed by MALDI-TOF-MS (AB SCIEX). Briefly, the crude products were analyzed by RP-HPLC to reveal the presence of a single main peak, which was isolated by preparative HPLC on a Vydac C18 Column (50 × 250 mm; 10 μm) (Western Analytical). The homogeneity of the final peptides was ascertained by analytical RP-HPLC, using a Vydac C18 column (4.6 × 150 mm; 5 μm), eluted with a H_2_O/0.1% TFA (A) and CH_3_CN/0.1%TFA (B) linear gradient, from 5 to 70% B over 30 min, at 1 mL/min flow rate. The identity of the products was confirmed by MALDI-TOF mass spectrometry, which gave the expected molecular weight.

### 3.4. E2-BS1 Cloning, Expression and Purification

The pETHBSE2 was constructed from pETHE2DISP [55,56] to allow the expression of the mimotope BS1 as *N*-terminal His tag fusion to the E2DISP core scaffold. Oligonucleotide sequences SEQ1: 5′TCCCCCCGGGCTGGGTGTTCACGCTGGTGTTCGGACCGTTCGGTTCCAGTTTCAC ACCACCGTACATCGCTTTACC3′ and SEQ2: 5′GGAATTCCATATGCCTATCAAACAGATC AACAACATGAGCCAGAAAGTGGGTAAAGCGATGTACGGTGGT3′ encoding the mimotope sequence RIKQINNMSQKVGKAMYGGVKLEPNGPNTSVNTQ was used to generated pETHBSE2.

These oligonucleotides include the sequences recognized by the restriction enzymes *Nde*I and *Xma*I (Biolabs). Briefly, the vector pETHE2DISP was digested with restriction enzymes *Nde*I and *Xma*I. The pairs of sequences SEQ1 and SEQ2 were annealed, digested by *Nde*I/*Xma*I, and inserted into the digested pETHE2DISP by ligation with T4 DNA ligase (Biolabs). Circular plasmid was selected on LB medium plates containing ampicillin using *E. coli* TG1 cells. The vector pETHBSE2 was purified using NucleospinPlasmid kit (QIAGEN) and successful construction of the plasmid was confirmed by DNA sequence analysis. His tag BS-fusion proteins were purified as described in [68] with slight modifications. Briefly, one liter of LB media containing 100 μg/mL ampicillin was inoculated with 10 mL of an overnight culture of *E. coli* BL21 (DE3) pETHBSE2 and maintained at 37 °C until an optical density of 0.6 (600 nm) was obtained. The cells were then heat-induced overnight at 30 °C with 1 mM IPTG. The bacterial cells were harvested by centrifugation (6000× *g* for 10 min) and the cell pellet was resuspended in 70 mL of 20 mM Tris-HCl pH 8.0, 10 mM EDTA, 1 mM PMSF (buffer A) containing 0.1 mg/mL lysozyme and placed in ice for 20 min. The cells were then lysed by sonication with short bursts (15 s on and 90 s off for 10 cycles, 14,000 Hz) and the insoluble inclusion bodies containing HBSE2 protein were recovered by centrifugation (10,000× *g* for 1 h) and were resuspended in 20 mL of 100 mM NaH_2_PO_4_, 10 mM Tris-HCl pH 8.0 (buffer B) containing 6 M GuHCl. The resuspended inclusion bodies were gently shaken on ice for 1 h and insoluble material was removed by centrifugation (10,000 × *g* for 15 min). Two millilitres of Ni-NTA agarose (Invitrogen) (previously washed with 2 times with 10 mL water and equilibrated with 10 mL of buffer B) was added to the clarified supernatant that was gently shaken overnight on ice. The mixture was then loaded into an empty column, and unbound protein was eluted with aliquots of wash buffer (buffer B) pH 6.3 until an optical density of 0.005 (280 nm) was obtained. The bound proteins were eluted with 2 × 5 mL aliquots of buffer B pH 5.9 followed by 2 × 5 mL of buffer B pH 4.5. The fractions were examined by SDS-PAGE (12% acrylammide), appropriate fractions were pooled and dialyzed against 2 L of buffer B without 6 M GuHCl for five times. The clarified supernatant containing HBSE2 was obtained by centrifugation (10,000× *g* for 15 min). The identity of HBSE2 protein was confirmed by Western blot performed with rabbit polyclonal antibody against E2 protein or with mouse serum containing antibodies against BS mimotope. The concentration of E2 constructs was determined by Coomassie dye binding method (Bradford assay). The purified protein samples were stored at −80 °C.

### 3.5. Electron Microscopy of E2-BS1 Complex

The electron microscopy was performed by placing a drop of protein solution (0.1 mg/mL) on a perforate carbonate film and removing the excess with a filter paper. The sample was then stained with 1% uranyl acetate solution. Molecules were photographed using an electron microscope PHILIPS EM208S at a magnification 160,000×.

### 3.6. Immunization Schedules in Mice

Six five weeks Balb/c females were divided in two immunization groups: the first group of three mice was immunized i.p. with 100 μg of E2-BS1; the second group was immunized i.p. with 100 μg E2 alone as a control. Antigen-adjuvant mixtures were prepared as follows: all the immunization antigens were brought to a final volume of 0.4 mL with PBS and an equal volume of complete Freund’s Adjuvant (cFA) for the first immunization; the incomplete adjuvant (iFA) was used in the remaining immunizations. A pre-immunization bleeding was collected as negative control from each mouse. All mice were immunized four times at 2 weeks interval and two bleedings samples were collected after the third and the fourth immunizations. Bleedings were assessed in ELISA for the specific antibody response against BS1, along with envelope glycoproteins m-gp120 SF162 and o-gp140ΔV2 SF162.

### 3.7. Immunization Protocols in Rabbits

To generate rabbit immunogen-specific polyclonal sera with anti-HIV-1 neutralizing activity, 10 New Zealand rabbits (five males and five females) were divided in two immunization groups. The first group was composed of six rabbits (three males and three females) all immunized with E2-BS1 antigen; the second group of four rabbits (two males and two females) was immunized with E2 protein as a control. Antigen-adjuvant mixtures were prepared as follows: all the immunization antigens were brought to a final volume of 1 mL with PBS and an equal volume of complete Freund’s Adjuvant (cFA) for the first immunization; the incomplete adjuvant (iFA) was used in the remaining immunizations. A pre-immunization bleeding sample was collected at day 0 then four boosting with equal dose of antigens (200 μg/500 μL every immunization for each rabbit) were performed i.m. and s.c. Three additional bleeding samples were collected at day 35, day 63 with the terminal bleeding at day 76, fourteen days after the fourth immunization.

### 3.8. ELISA

Direct enzyme-linked immunoassorbent assays (ELISAs) were used to detect BS3 and E2-BS1 specific IgG reactivity. 96-wells Maxisorp microtiter plates (NUNC) were coated with 100 μL/well containing 100 ng of purified BS3 peptide mimotope or, alternatively, 2 μg of E2-BS1 complex in PBS 1× and incubated at 4 °C O/N. The following day, the plates were washed and blocked in 200 μL of blocking buffer (PBS 1×, 5% non-fat dry milk, 0.01% tween 20). Serum samples, diluted 1:1000 in blocking buffer, were incubated for 2 h at 37 °C, washed six times with PBS 1× containing 0.05% tween 20 and then incubated with a 1:5000 dilution of Alkalyne Phosphatase conjugated goat anti-human IgG Fc specific (SIGMA, St. Louis, MO, USA) for 1 h at 37 °C. After extensive washing, samples were incubated with PNPP (Para-Nitro-Phenyl-Phosphate) platelets substrate diluted in Diethanolamine substrate solution pH 9.6 (10 min at room temperature). Antibody titers specific for BS3 or m-gp120 SF162 and o-gp140ΔV2 SF162 were measured in standard ELISA assay in which m-gp120 SF162 and o-gp140ΔV2 SF162 were coated overnight at 100 μL/well each at a concentration of 5 μg/mL. After 2 h blocking at 37 °C, linear increasing sera dilutions (1:100–1:1,000,000) were incubated for 3 h at 37 °C with the envelope antigens. Colour development was read twice at 420 nm on a MULTISCAN EX ELISA Plate reader (Thermo Labsystems, Franklin, MA) after 20 and 40 min from the last incubation time. Results were evaluated as fold increase (ratio between the optical density (OD) of each diluted sample and the OD of both the donor serum and pre-immunization sera). Endpoint titers for immunized mice and rabbits were established as the last dilution with a fold increase ≥3.

### 3.9. Competitive ELISA Assay

A competitive ELISA assay was performed as follows: 96-wells Maxisorp microtiter plates were coated overnight at 4 °C with 100 μL/well m-gp120 SF162 at a molar concentration of 1 nM in PBS 1×. During 2 h blocking at 37 °C as described above, 5 pre-incubation mix were prepared by using the 5/76 rabbit serum (dilution 1:2000), previously tested reactive with m-gp120 SF162, or 5/76 pre-immunized rabbit serum (control) and linear increasing molar concentrations (1 nM–10 μM) of E2-BS1 complex or BS3 synthetic peptide mimotope. A dilution 1:2000 with fold increase of 5 was chosen to clearly show the serum reactivity against m-gp120 SF162. Pre-incubation mix was incubated 2 h at room temperature on a mechanic wheel. Then, mix was incubated overnight at 4 °C to improve the antibody binding to the antigen. After extensive washing to eliminate all unspecificities and 1 h of incubation with Alkalyne phosphatase conjugated antibody, substrate solution was added and the plates were analyzed as described above. Colour development was read twice at 420 nm on a MULTISCAN EX ELISA Plate reader (Thermo Labsystems, Franklin, MA) after 20 and 40 min from the last incubation time. Results were evaluated as fold increase.

### 3.10. Measurement of Neutralizing Response

E2-BS1 immunized rabbits sera, tested positive for the antibody response against m-gp120 or o-gp140ΔV2 SF162, were assessed for the presence of vaccine-induced neutralizing antibody by using a neutralization assay based on reductions in luciferase (Luc) reporter gene expression after a single round of virus infection with pseudotyped HIV-1 viruses in highly infective TZM-bl cells performed as described [62]. Neutralizing antibody levels in the sera of rabbits immunized with E2-BS1 and E2-wt or pre-immunized rabbit sera as controls were measured against a panel of three pseudotyped HIV-1 viruses: SF162.LS, NL-ADArs belonging to clade B and one primary isolate HIV-0012466-2.52 belonging to clade C. In this assay, 200 TCID_50_ of virus was incubated with diluted samples in triplicate in a total volume of 150 μL for 1 h at 37 °C in 96-well flat-bottom culture plates. Freshly trypsinized cells (10,000 cells in 100 μL of growth medium containing 75 μg/mL DEAE dextran) were added to each well. One set of control wells received cells plus virus (virus control) and another set received cells only (background control). After 48 h incubation, 100 μL of cells were transferred to a 96-well black solid plate (Costar, Corning, NY, USA) for measurements of luminescence using Bright Glo substrate solution, as described by the supplier (Promega, Madison, WI, USA). The percent neutralization was calculated by comparing experimental wells to virus control wells. Neutralization titer was evaluated as the dilution at which RLUs were reduced by 50% compared to virus control wells after subtraction of background RLUs using pre-bleed sera.

### 3.11. Statistical Analysis

The standard parametric Student’s *t* test was used to analyze the differences of ELISA results between HIV^+^ sera (LTNPs and AIDS) and HIV^−^ donor serum and between E2-BS1 and E2-control immunized mice or rabbits groups. The standard parametric Student’s *t* test was also used to analyze the differences of rabbit sera/m-gp120 SF162 binding displacement between the competitors BS3 and E2-BS1 at the same molar concentrations. Standard deviation was calculated for all ELISA experiments. All tests were performed by using both EXCEL and the Statistic program GraphPad.

## 4. Conclusions

The extreme flexibility, the sequence hypervariability and the glycosylation pattern of the gp120 are the major problem in eliciting an effective vaccine that counteracts the HIV-1 infection [25,26,69–72]. Significant efforts have gained a deep understanding of neutralization epitopes together with their interaction with neutralizing antibodies [39–50]. However, although substantial effort has focused on the design of immunogens capable of eliciting antibodies *de novo* that would target similar epitopes [73–75], it remains uncertain whether a conventional vaccine will be able to elicit analogues of the existing broadly neutralizing antibodies.

We adopted a hybrid vaccine strategy based on creating and modeling by bioinformatics programs [76,77] discrete and highly conserved discontinuous epitopes recognized by well-known monoclonal antibodies or HIV^+^ patient sera and by transplanting them into highly immunogenic E2 protein scaffold [70]. We focused on the bridging sheet domain of gp120 env protein because it contains amino acidic sequences essential for the CD4 and the coreceptor binding, together with V1/V2 and V3 hypervariable regions [27,31,33,77,78]. Upon an extensive bioinformatics analysis, we devised four structurally constrained peptide mimics of the bridging sheet structure: we assembled linear peptides composed of four antiparallel β strands linked to each other by mini-loops GG or NGP sequences replacing V1/V2 loop sequence between β2 and β3 strands and the short loop between β20 and β21. Further, to overcome the high hydrophobicity, we introduced amino acidic substitutions in the peptide mimotope sequence to improve its water solubility. Two residues of β19 proved crucial for a correct bridging sheet refolding and were added in the sequence of mimotope BS3. As amino acidic substitutions in β20 and β21 strands alter the co-receptor binding site, we reproduced the two additional mimotopes BS1 and BS2 with modified β2, β3 and β20 but unmodified β21 strand amino acidic sequence. Molecular modeling programs showed that mimotopes refolded as the original bridging sheet. To display the BS1 mimotope, it was directly transplanted on the surface of the E2 prokaryotic scaffold of the *Geobacillus stearothermophylus* PDH complex. The choice of E2 multimeric subunit allowed the complex structural refolding as a 20-trimer which is a suitable carrier for the mimotope BS1 presentation to the immune system, since it could mimic the trimeric structure of Env spike. Previous studies on E2 protein demonstrated its efficiency to elicit both humoral and cellular immune response against the epitope exposed on its surface [53,79,80]. Before testing the immunogenic properties of mimotope BS1 exposed on the arm of core domain of E2 protein, we performed ELISA assays to assess the reactivity of synthetic mimotopes BS3 and BS4, together with the reactivity of E2-BS1 complex against HIV^+^ patient sera (LTNPs and AIDS conclamate). The reactivity of E2-BS1 complex against HIV^+^ sera was significantly higher than the reactivity of chemically synthesized mimotopes to demonstrate that BS1 amino acidic sequence fused to core domain of E2 protein was a good structural mimotope of natural bridging sheet. This result, together with electron microscopy and western blot analysis, validated the E2 sixty-mer as a suitable carrier to expose an antigen to the immune system. Firstly, the immunogenicity of E2-BS1 complex was evaluated in mice. In the presence of high antibodies titers elicited against the BS1 mimotope, no cross-reactivity with the envelope was shown, suggesting the use of rabbits for further immunization experiments. Indeed, rabbits were selected as the only rodents producing antibodies with a long protruding CDR3 hypervariable region [81] and able to recognize epitope structures of self-antigens, including cardiolipin and HIV-1 neutralizing epitopes recognized by human HIV-1 neutralizing antibodies, such as IgG1b12, mAb17b, mAb 2F5, mAb 4E10 [82–87]. Rabbit immunizations by using two different immunization sites, i.m. and s.c., produced antibodies that specifically bound both m-gp120 SF162 and o-gp140dV2 SF162, thus proving the evolutive advantage of rabbits *versus* mice as animal models to be used in immunization experiments. The antibodies specificity was finally tested by performing a competitive ELISA assay with progressively increasing concentrations of BS3 mimotope and E2-BS1 complex as competitor. Both the BS3 mimotope and the E2-BS1 complex displaced the rabbit serum binding with m-gp120 SF162 in a dose dependent manner even if E2-BS1 was the best competitor. These results further explained that the BS1 linked to the core domain of E2 subunit could antigenically mimic the relative bridging sheet epitope on the HIV-1 envelope. The last experiment to definitively demonstrate the possibility to elicit strong and broadly neutralizing antibodies was conducted by using highly infectious TZM-bl cells expressing both CCR5 and CXCR4 and a panel of three viruses (two highly neutralization sensitive viruses belonging to western clade B, SF162.LS and NL-ADArs, and one primary isolate belonging to African clade C, HIV-0012466-2.52). Although positive neutralizing activity against all the three viruses was shown in one animal, the antibody titer able to neutralize the HIV infection *in vitro* was milder for the HIV clade C primary isolate than the highly sensitive clade B viruses.

Here, we have reported the first evidence of mild neutralizing antibodies elicited in rabbits against HIV primary isolate by using a linear reconstituted bridging sheet mimotope fused to a carrier protein. To date, all the attempts to reproduce the bridging sheet [28,31,88], a highly conserved region hidden inside the envelope spike, have failed. We also confirmed that E2 is a good immunogenic carrier [54,80] exposing the bridging sheet in a correct antigen structure. The mild neutralization titers reported in this work may provide a rationale for further investigations into the bridging sheet accessibility on the viral envelope, for example by engineering other envelope variants in a CD4-bound state, pre-fusogenic intermediate or by mutagenizing our mimotope to obtain a more constrained conformation able to be recognized both by CD4i antibodies and by HIV^+^ sera.

Neutralizing antibodies are thought to be crucial for HIV vaccine protection; however, studies in animal models suggest that high antibody concentrations are required. This is a major hurdle for vaccine design. These studies typically rely on a large virus inoculum to ensure infection in control animals in single-challenge experiments. In contrast, most human infection via sexual encounters probably involve repeated exposures to much lower doses of virus. Therefore, animal studies may provide an over-estimated level of antibodies required for protection in humans [89]. A recent study demonstrated that lower amounts of antibodies may protect macaques from repeated intravaginal exposure to low doses of a simian immunodeficiency virus-HIV chimera (SHIV) [89]. Moreover, the antibody neutralizing response against HIV-1 env conserved epitopes could be improved by using a heterologous DNA priming-protein, boosting immunization strategies. Finally, finding neutralization epitopes in conserved envelope regions different from classical immunodominant epitopes of hypervariable regions, could underscore novel vaccine candidates able to elicit a strong specific antibody response at early stages.

## Figures and Tables

**Table 1:** End point titration against o- and m-Env of IgGs purified from mice and rabbit sera. Three indirect ELISA assays were performed to assess the cross-reactivity of mice and rabbits sera from animals immunized with E2-BS1 against monomeric (m) and oligomeric (o) gp120. The table reports end-point titers against BS1 mimotope, E2 protein carrier, m-gp120 SF162 and o-gp140ΔV2 SF162 for both mice and rabbits. Statistically significant results for the reactivity against m-gp120 SF162 and o-gp140ΔV2 SF162 are indicated.

		ELISA Antigen Titer after Four iImmunizations *			
		
Animal a	Immunogen	Bridging Sheet	gp120-SF162	gp140DV2-SF162	E2 Protein
Mouse1	E2-BS1	100,000	<20	--	500,000
Mouse2	E2-BS1	100,000	<20	--	1,000,000
Mouse3	E2-BS1	50,000	<20	--	500,000
Mouse4	E2	<20	<20	--	1,000,000
Mouse5	E2	<20	<20	--	1,000,000
Mouse6	E2	<20	<20	--	500,000
Rabbit1 (#E2-BS60 male)	E2-BS1	500,000	400 b	--	1,000,000
Rabbit2 (#E2-BS60 female)	E2-BS1	2000	200 b	--	500,000
Rabbit3 (#E2-BS male)	E2-BS1	500,000	400 c	--	1,000,000
Rabbit4 (#E2-BS female)	E2-BS1	150,000	20,000 c	--	1,000,000
Rabbit5 (#5/76)	E2-BS1	100,000	5000 c	3000 c	500,000
Rabbit6 (#6/76)	E2-BS1	100	<20	<20	75,000
Rabbit7 (#1/76)	E2	<20	<20	<20	500,000
Rabbit8 (#3/76)	E2	<20	<20	<20	1,000,000
Rabbit9 (#E2-wt female)	E2	<20	<20	--	1,000,000
Rabbit10 (#E2-wt male)	E2	<20	<20	--	500,000

* Values indicate relative endpoint ELISA titer against the indicated proteins;

a Sera were collected two weeks following the fourth immunization (day 76);

-- Non-detected;

b Values are statistically significant with *p* < 0.01;

c Values are statistically significant with *p* < 0.05.

**Table 2:** IgGs neutralization assay from rabbits immunized with E2-BS1. (**A**) Two sets of neutralization experiments with HIV-1 infected TZM-bl cells were performed to assess the ability of immunized rabbit IgGs to neutralize the infection of two clade B strain viruses: SF162.LS and NL-ADArs, along with one clade C HIV-0012466-2.52 primary isolate. ID50 (dose of virus infecting the 50% of cells) was calculated by using increasing sera dilutions. Dilutions ≥30 were positive for neutralization activity. In all the experiments the rabbit 5/76 showed a mild neutralization titer against all three viruses tested. (**B**) Evaluation of the other immunized rabbits showed no neutralization titer against the three clade B strain viruses: SF162.LS; HIV-1 MN; SIVmac239CS.23. (*p* < 0.05).

A				

		ID50 in TZM-bl Cells 1		
		
Animal (rabbits)	Bleed	SF162.LS	HIV-0012466-2.52	NL-ADArs
**First set of assays**				
Rabbit 5	post-immune	114	30	64
Rabbit 8	post-immune	27	28	22
**Second set of assays**				
Rabbit 5	post-immune	109	53	155
Rabbit 8	post-immune	<20	30	<20
naive rabbit pool	Naïve	<20	<20	<20

**B**				

		**ID50 in TZM-bl Cells** 1		
		
**Animal (Rabbits)**	**Bleed**	**HIV-1 MN**	**SF162.LS**	**SIVmac239CS.23**

Rabbit 9	Post-immune	<20	<20	<20
Rabbit 10	Post-immune	<20	<20	<20
Rabbit 1	Post-immune	<20	<20	<20
Rabbit 2	Post-immune	<20	<20	<20
Rabbit 3	Post-immune	<20	<20	<20
Rabbit 4	Post-immune	<20	<20	<20

1 Values indicate the sample dilution at which the relative luminescence units (RLUs) were reduced by 50% as compared to virus control wells (no test sample).
